# Missile embolism from pulmonary vein to left ventricle: report of a case

**DOI:** 10.3389/fcvm.2024.1342146

**Published:** 2024-02-23

**Authors:** Mohammadrafie Khorgami, Fattaneh Khalaj, Maziar Gholampour, Hassan Tatari

**Affiliations:** ^1^ Rajaie Cardiovascular Medical and Research Center, Iran University of Medical Sciences, Tehran, Iran; ^2^ Liver and Pancreatobiliary Diseases Research Center, Digestive Diseases Research Institute, Tehran University of Medical Sciences, Tehran, Iran

**Keywords:** missile embolization, gunshot, left ventricle, pulmonary vein (PV), foreign body

## Abstract

Missile embolization is rare in penetrating trauma, occurring in 0.3% of cases. Bullet embolism into the left ventricle is less frequent, with few instances described in the literature. This paper describes an instance of left ventricular bullet embolism from the pulmonary venous system following gunshot chest trauma. A 7-year-old boy sustained a gunshot wound to his chest during an assault accident. Despite thoracic pain, he remained conscious and exhibited vital signs. A CXR and CT scan revealed a bullet in the left mediastinum. A left thoracotomy was performed to remove blood and clots from the pericardium. The patient was sent to a tertiary referral hospital for further investigation. The patient underwent elective surgery to remove the foreign body from inside the heart. The procedure involved a partial thymectomy and pericardial opening, and the patient was released from medical care after 14 days. After 6 months, there were no signs or symptoms of cardiothoracic infection or evidence of mitral valve regurgitation in echocardiography.

## 1 Introduction

Missile embolization is a rare occurrence in penetrating trauma, where an object enters the bloodstream and travels to another part of the vascular system. In fact, according to a review of over 7,500 casualties from the Vietnam War, bullet embolization occurred only in 0.3% of cases (1). Although reports suggest emboli rarely occur in the pulmonary vasculature and right heart, bullet embolism into the left ventricle following penetrating chest injuries is considerably less frequent with few instances described in the literature (2–7). Due to a lack of extensive experience at any single institution, there are several controversies surrounding appropriate diagnostic and therapeutic approaches for managing such cases. This paper describes an instance of left ventricular bullet embolism from the pulmonary venous system following gunshot chest trauma.

## 2 Case description

**Figures 1, 2:**
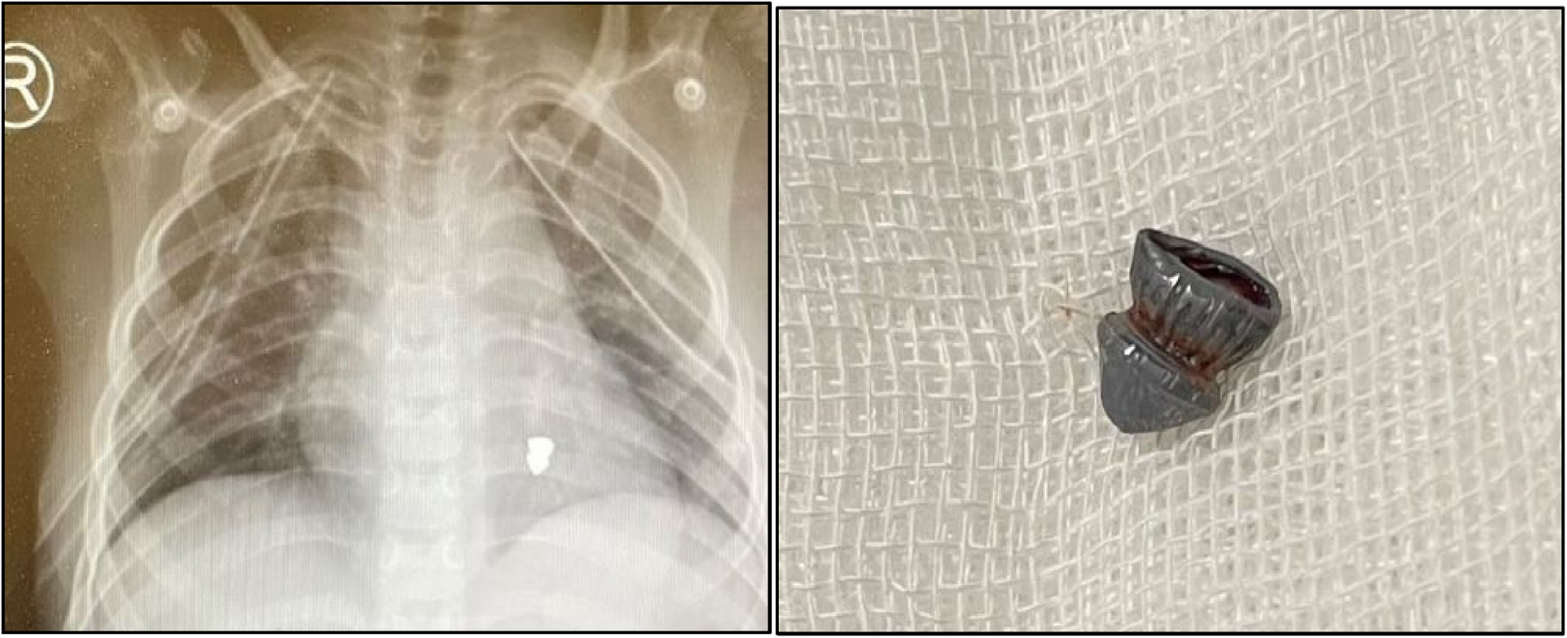
According to the CXR, a bullet of 5.5 mm in length was found in the anteroposterior of left part of the mediastinum, gunshot.

**Figures 3, 4:**
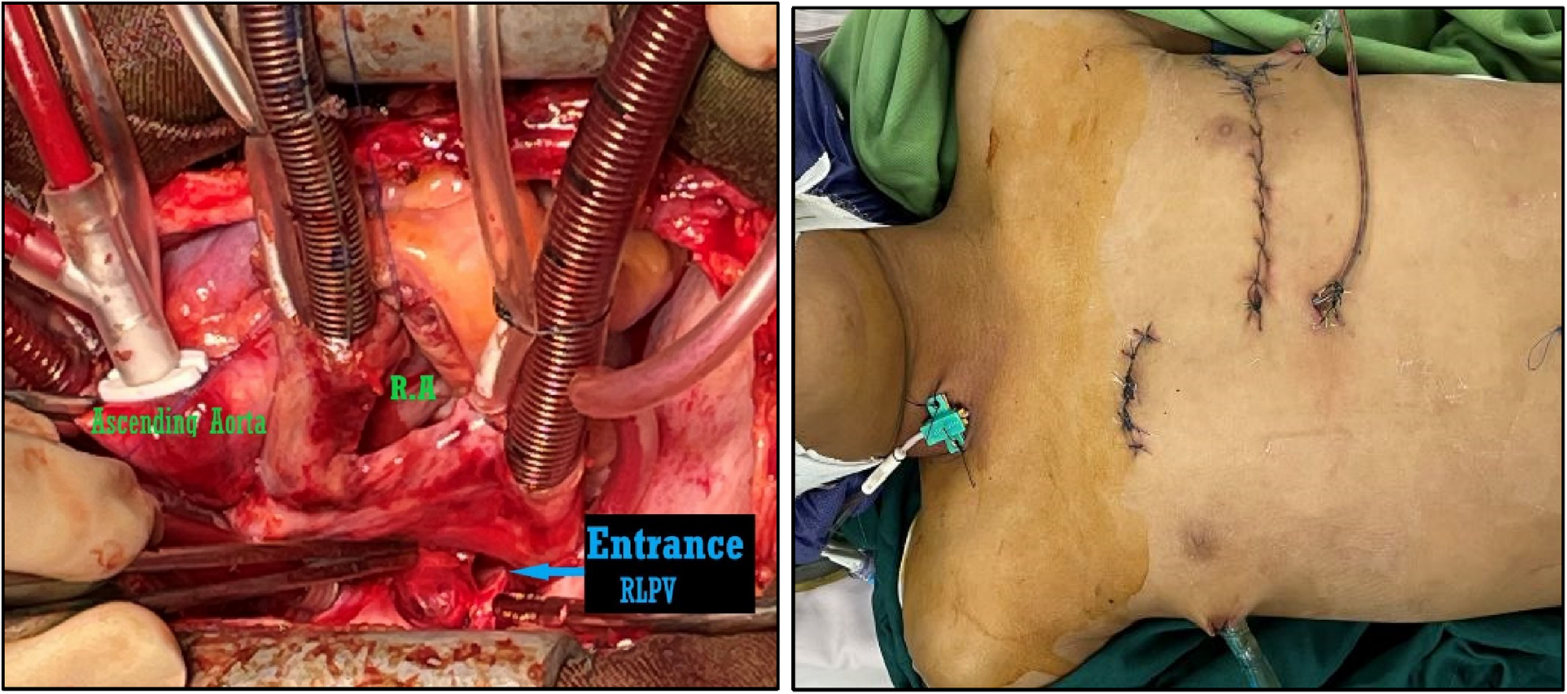
The entrance location of the bullet was discovered after right atriotomy and septostomy in the anterosuperior portion of the lower right pulmonary vein, and the bullet that had been lodged in the posterior chordal lobe of the mitral valve was removed and chest wall was repaired.

The presented case report describes a 7-year-old boy without history of other medical concerns who was brought to the emergency unit after sustaining a single gunshot wound to the right side of his chest, resulting from an assault accident while on his way to school. There was no exit wound. Despite complaining of thoracic pain, the boy remained conscious and exhibited vital signs, including a blood pressure of 116/79 mm Hg, a pulse rate of 118 beats per min, and a respiratory rate of 38/min. After examination by a general surgeon, a chest tube was placed in the damaged thorax (right), and a small amount of blood and air were released. Further examination following the relative stability of hemodynamics revealed an entrance wound near the right side of the sternum at the level of the third intercostal space. There was no marked subcutaneous emphysema or jugular venous distention. His initial hematocrit was 32%. Arterial blood gases did not detect hypoxemia or acidosis, and an electrocardiogram showed normal sinus rhythm. Upon conducting a chest radiograph (CXR) and computerized tomography (CT) scan, it was determined a bullet (5.5 mm long) was in the left part of the mediastinum (Figures 1, 2). Therefore, a pleuropericardial window is installed through the left thoracotomy, which removes some blood and clots from the pericardium. After completing these procedures and receiving intravenous fluids, the patient's vital signs became completely stable, and he was referred to the local hospital with a cardiologist for further investigation, where echocardiography was performed and placing the bullet in the left ventricle was confirmed. Considering the risk of embolization, the patient is sent to the tertiary referral hospital. Transesophageal echocardiography (TEE) showed that the right atrium and ventricle were functioning normally, with a thick particle in the left ventricle (LV) cavity behind the posterior leaflet and mild mitral regurgitation (MR). No aortic insufficiency (AI) or pulmonary vein (PV) stenosis were also observed. In addition, high-resolution computed tomography (HRCT) revealed left pleural effusion, scattered sub-segmental atelectasis in both lungs, and bilateral mild pneumothorax. Due to the possibility of bullet embolization, the patient underwent elective surgery to remove the foreign body from inside the heart. The surgical procedure was conducted via the median sternotomy approach. A partial thymectomy and pericardial opening were performed. The quantity of blood and clots evacuated from the pericardium Although dissection of the mediastinum and the great vessels was challenging, it was executed without harm. The entry point of the bullet was found in the anterosuperior part of the lower right pulmonary vein, which was covered by a clot. After the right atriotomy, the interventricular septum was split, and the bullet stuck in the posterior chordal lobe of the mitral valve was released and removed (Figure 3). Then the septum between the atrium and right atrium and the pulmonary vein are damaged and repaired, and after coronary perfusion is established and normal heart contractions return, cardiopulmonary bypass is stopped and the chest wall is repaired (Figure 4). Following the closure of the sternum, the patient was transferred to the intensive care unit and was deemed to be in stable condition. The patient underwent extubation within the initial 24 h following the surgical procedure. The individual achieved full recuperation and was released from medical care after 14 days. During a subsequent 6-month evaluation, the patient continued to exhibit a state of well-being, and there were no symptoms or signs of cardiothoracic infection or evidence of MR in echocardiography.

## 3 Discussion

Missile embolism is a rare subtype of foreign body embolization that can occur as a complication of gunshot injuries. In such cases, the missile enters the vessel or cardiac cavity but loses its kinetic energy and remains within the lumen instead of passing through. Embolization becomes a concern when there is no exit wound for the projectile, and it can be located distant from its expected trajectory. While most instances occur shortly after injury, delayed occurrences ranging from days to years have also been reported in the literature (8, 9). The incidence of this phenomenon has been reported to be 0.3% in the Vietnam War and 1.1% in more recent conflicts, such as the Afghanistan and Iraq Wars. However, its occurrence in civilian populations remains unknown, although it may be higher than in military settings due to the lower velocity and kinetic energy of civilian weapons (1, 10, 11). As previously mentioned, most venous emboli migrate with the direction of blood flow and are typically found in the pulmonary arterial system or right heart (12). Missile emboli to the left heart are infrequent and occur via direct injury, entry through the pulmonary venous system, or a patent foramen oval (13). Symbas conducted a literature review spanning from 1940 to 1988 and found that out of the 201 patients with retained missiles in their hearts, the majority (87%) were due to direct injury to the heart or pericardium. The remaining 13% (*n* = 27) were caused by an embolization originating from a distant site of injury. Of those, 81% had an injury occur within a systemic vein resulting in embolization to the right heart, while only one patient experienced damage to a pulmonary vein leading to embolization on the left side of the heart. For the remainder of the cases studied, it was unclear where exactly entry into circulation occurred (12). According to Advanced Trauma Life Support principles, stabilization is crucial in penetrative chest trauma. This involves performing life-saving interventions such as endotracheal intubation, placement of chest tubes, transfusion of blood products, or emergency department thoracotomy. In an unstable patient, even during the initial assessment, a chest x-ray study is vital because it provides information concerning the location and potential trajectory of a projectile if present, which can determine if there is the absence or presence of pneumothorax or hemothorax. The presence of a blurred appearance around the heart on an x-ray indicates a movement that may indicate intracardiac or pericardial positioning (14). Patients with stable hemodynamics presenting with projectiles within their hearts pose diagnostic and therapeutic challenges that require accurate localization and evaluation for associated injuries using multidisciplinary approaches, including surgical intervention radiographic analysis, endoscopy, or bronchoscopy. Using a CT scan represents an ideal imaging modality to localize projectiles accurately and ensure evaluation for associated injuries, including pulmonary parenchyma injury assessment and thoracic vascular structures assessment; whereas tracheobronchial tree damage on top esophagus injury judging is possible with this method (15, 16). In cases where there are apprehensions regarding injuries to the aforementioned structures, it is recommended to employ esophagoscopy or esophagogram and bronchoscopy to evaluate the esophagus and tracheobronchial tree, respectively. An intracardiac projectile can be localized through various modalities, such as TTE (transthoracic echocardiography), TEE, fluoroscopy, and coronary angiography. TTE is useful for unstable patients, as it can quickly confirm or rule out the presence of a pericardial effusion that may require operative intervention (17). However, TEE is superior to TTE in determining the precise location of the projectile and the extent of myocardial damage, injury to valves, or septum. Fluoroscopy may be helpful when echocardiography fails to determine bullet location accurately (18–20). The control of intracardiac bullets in stable patients is a topic of debate within the medical community due to the challenges in predicting long-term outcomes, which can vary from asymptomatic to severe and life-threatening. The occurrences related to a projectile are influenced by various factors such as the site, size, shape, and mobility. it is imperative to tailor treatment to the specific characteristics of the projectile (21) for instance, Nagy et al. expressed that tiny projectiles measuring 5 mm, situated bilaterally adjacent to the heart, characterized by their smooth contours and complete intramyocardial embedding, typically exhibit no symptoms and may be conservatively managed without intervention (22).

## 4 Conclusion

The removal of intracardiac missiles is not always necessary. A limited number of patients who present at medical facilities with vital signs after experiencing penetrating trauma and bearing a foreign object within the heart necessitate expeditious resuscitation and hemodynamic stabilization. Management strategies should be tailored to each specific case. If there are missiles that are completely embedded in the heart and do not cause any symptoms, they can be monitored without intervention. On the other hand, any missiles that cause symptoms should be removed. Missiles that are asymptomatic and located on the left side of the heart should be removed to prevent systemic embolization and potential peripheral or cerebral ischemia. Likewise, asymptomatic missiles on the right side of the heart should be removed to prevent pulmonary emboli if they are larger than 5 mm. However, if a right-sided missile has already embolized the pulmonary circulation but is located peripherally and the patient remains asymptomatic, it can be observed without intervention (23).

## Data Availability

The original contributions presented in the study are included in the article/Supplementary Material, further inquiries can be directed to the corresponding author.
